# A Web-Deployed, Explainable AI System for Comprehensive Brain Tumor Diagnosis

**DOI:** 10.3390/neurolint17080121

**Published:** 2025-08-04

**Authors:** Serra Aksoy, Pinar Demircioglu, Ismail Bogrekci

**Affiliations:** 1 Institute of Computer Science, Ludwig Maximilian University of Munich (LMU), Oettingenstrasse 67, 80538 Munich, Germany; serra.aksoy@campus.lmu.de; 2 Department of Mechanical Engineering, Aydin Adnan Menderes University (ADU), Aytepe, 09010 Aydin, Turkey; ibogrekci@adu.edu.tr

**Keywords:** brain tumor diagnosis, deep learning, volumetric segmentation, explainable AI (XAI), web-based platform

## Abstract

Background/Objectives: Accurate diagnosis of brain tumors is one of the most important challenges in neuro-oncology since tumor classification and volumetric segmentation inform treatment planning. Two-dimensional classification and three-dimensional segmentation deep learning models can augment radiological workflows, particularly if paired with explainable AI techniques to improve model interpretability. The objective of this research was to develop a web-based brain tumor segmentation and classification diagnosis platform. Methods: A diagnosis system was developed combining 2D tumor classification and 3D volumetric segmentation. Classification employed a fine-tuned MobileNetV2 model trained on a glioma, meningioma, pituitary tumor, and normal control dataset. Segmentation employed a SegResNet model trained on BraTS multi-channel MRI with synthetic no-tumor data. A meta-classifier MLP was used for binary tumor detection from volumetric features. Explainability was offered using XRAI maps for 2D predictions and Gaussian overlays for 3D visualizations. The platform was incorporated into a web interface for clinical use. Results: MobileNetV2 2D model recorded 98.09% classification accuracy for tumor classification. 3D SegResNet obtained Dice coefficients around 68–70% for tumor segmentations. The MLP-based tumor detection module recorded 100% detection accuracy. Explainability modules could identify the area of the tumor, and saliency and overlay maps were consistent with real pathological features in both 2D and 3D. Conclusions: Deep learning diagnosis system possesses improved brain tumor classification and segmentation with interpretable outcomes by utilizing XAI techniques. Deployment as a web tool and a user-friendly interface made it suitable for clinical usage in radiology workflows.

## 1. Introduction

Brain tumors are one of the most complicated and hazardous medical ailments one can experience. This is not only due to their inherent biological malignancy but also due to the diversity of issues they present within both the diagnostic phase as well as in the formation of effective treatment strategies. The symptomatology of brain tumors is typically one of non-specificity or vagueness, and this has significant implications for both patients who are experiencing symptoms and for medical doctors attempting to diagnose the problem. This non-specificity has the effect of rendering it extremely difficult to determine the underlying cause in the early phases of the disease process. Furthermore, the radiological features expressed by these tumors when imaged through magnetic resonance imaging (MRI) scans can display a high level of variability with regard to a large range of parameters, including but not limited to shape, size, location, and patterns of enhancement. This is another difficulty in what is already a complicated diagnostic process for brain tumors. Due to the many complexities that are inherently intertwined with the process, it must be understood that even the most experienced and skilled radiologists may find it quite challenging to interpret such scans accurately and in a timely fashion. This challenge is quite pronounced in high-pressure clinical environments where the stakes are astronomically higher and expectations placed on healthcare professionals are quite elevated [1,2,3,4,5].

MRI is the most common imaging technique used for detecting and diagnosing brain tumors. It owes its popularity to its greater capacity for offering better contrast in the instance of soft tissues, a factor that plays a key role in offering a clear distinction between the numerous structures found in the brain. Furthermore, MRI possesses the distinct and significant characteristic of not just providing very accurate structural details of many organs and tissues in the body but also providing useful functional details of physiological processes. It is its capability to provide both that renders it a very critical imaging modality in the fields of medical diagnosis and treatment planning, as indicated in studies by [6,7]. T1, T2, FLAIR, and contrast-enhanced T1-weighted imaging MRI sequences are important in medical imaging. They are important for a broad variety of activities for tumor detection, tumor grading, and chronic evaluation of the effectiveness of a treatment, along with the passage of time [8,9]. Yet, the intricate process of MRI scan interpretation has been largely described to be extremely time-consuming, usually necessitating an exceedingly high level of specialized skill and knowledge. The process’s sophistication makes it an extremely demanding task that is extremely prone to human error. This susceptibility to error is even more pronounced when attempting to handle the difficulty of tumor heterogeneity, in addition to navigating challenging anatomical structures, which can further complicate the interpretation process [10].

In order to efficiently overcome and transcend these various challenges that have been faced, deep learning (DL), and more specifically, convolutional neural networks (CNNs), has been the most successful and promising candidate in the field of neuroimaging. It has played a key role in bringing forth exceptional levels of performance in the pivotal tasks of detection, segmentation, and classification of tumors, as is evident from the studies by [11,12,13]. In addition to this, architectural frameworks based on the use of U-Net, DeepLabV3+, EfficientNet, and ResNet50 have seen widespread use and implementation in the specialized and complex domain of brain tumor analysis tasks. They achieve good performance on a series of benchmark datasets that have been long-standing and well-established in the research community over a protracted period. Such notable datasets include the BraTS dataset and the TCIA dataset, which have been extensively cited in research articles. The performance of these architectures has been expounded in lengthy research contributions, especially those by [14,15,16]. Naturally, in spite of these higher performance numbers, it ought to be mentioned that these models are typically black boxes. That is, they can make predictions without granting any sort of explanation or insights regarding how they reached their outputs, which can be a serious drawback in their actual applications in the real world. The unexplainability of these kinds of systems can lead to an immense number of issues and complications in clinical setups, where accountability and interpretability are paramount and of utmost importance [9,13].

The lack of transparency in this area has, in a substantial way, greatly contributed to an overwhelming increase in curiosity and interest regarding the developmental path and future direction of explainable artificial intelligence, which is often referred to by the acronym XAI. This area of study is quite recent and is dedicated to the important task of explaining and depicting the complex decision-making mechanisms that are at the core of deep learning models. This endeavor has been stressed and acknowledged by several high-profile researchers working in this domain, including some of the leading names like [17,18]. XAI techniques, including Grad-CAM, SHAP, LIME, SmoothGrad, and integrated gradients, possess the ability to generate many visualizations. These can be in the format of heatmaps and attribution maps, which are essential functions of model decision analysis. Most importantly, these kinds of visual representations are effective in accurately locating the areas of the input image that have contributed significantly towards the output decision made by the model [10]. The image reports that are being considered are very important, especially with respect to the process of verifying the claim that the model in question is concentrated on precisely those areas that are of clinical relevance and concern. For instance, it gives major emphasis to important features, such as the outlining of the edges of enhancing tumors, rather than indicating towards tissues that have no significance for the clinical evaluation in question [16].

Over the past few years, a variety of rigorous frameworks infused with explainable artificial intelligence (XAI) have been proposed to effectively bridge the prevailing interpretability gap that has been a concern. A notable framework in this context is NeuroXAI, which is highly commendable as it accommodates a range of backpropagation-based XAI methods and provides 2D and 3D visualizations specifically designed for classification and segmentation tasks, as presented in the work of Zeineldin et al. [9]. Continuing in this direction, Saeed et al. [13] present an original and reliable multi-phase pipeline known as Neuro-XAI, which seamlessly integrates DeepLabV3+, employs Bayesian hyperparameter optimization, and applies entropy-based uncertainty estimation, all with the aim of generating results that are not only reliable but also easily interpretable and explainable. Mahesh et al. [16] take the research to the next level by proving that if the Efficient-NetB0 model is judiciously coupled with the Grad-CAM approach, a classification accuracy of over 98% can be achieved. Not only is this exceptional integration enhancing the overall accuracy of the model, but it also produces interpretable visual diagnostic saliency maps that are consistent with clinical expectations.

With advancements in technology, regulatory demands such as the EU’s General Data Protection Regulation (GDPR) now demand interpretability of algorithms in clinical decision-making systems. This also calls for the construction of interpretable AI systems that are transparent and can be verified by clinicians, especially in life-critical applications such as neuro-oncology. Explainable deep learning systems represent a very significant and valuable advancement in the field of medical image analysis. Not only do such advanced systems greatly improve diagnostic performance and therefore generate more accurate and reliable results, but they also go a great way toward building and sustaining trust among clinicians who rely on these technologies. In addition, they sufficiently meet increasing ethical and legal demands for transparency so that the operations and decisions underlying their analyses are understandable and clear to users. This research seeks to optimize and greatly enhance the diagnosis of brain tumors, specifically focusing on using MRI technology in an effective way.

## 2. Materials and Methods

### 2.1. Data Acquisition and Preprocessing

**Figure 1:**
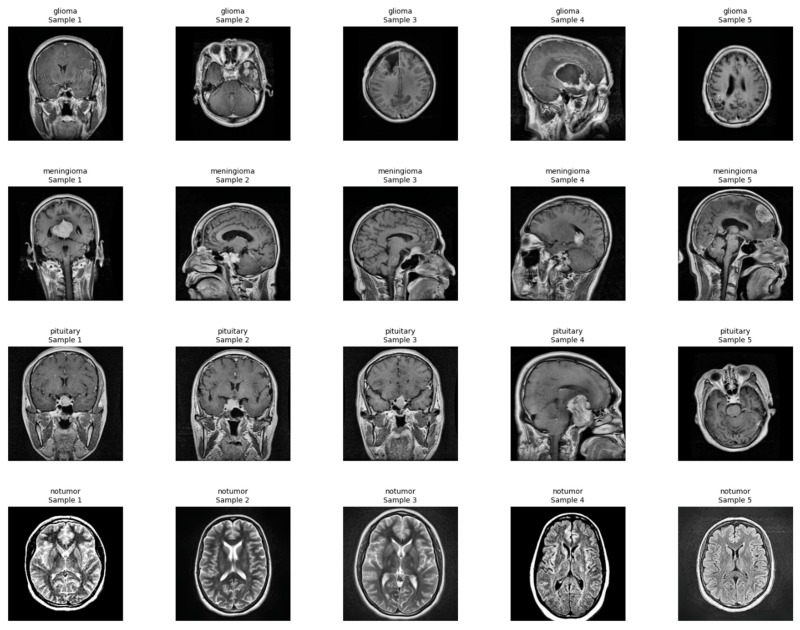
Random samples from each class in the 2d classification dataset.

The two independent but complementary brain tumor datasets from MRI scans were utilized in this study for the classification and segmentation tasks, respectively. The first dataset was utilized for the task of 2D classification of the type of tumor, while the second one was utilized for 3D segmentation of the tumor. Both datasets were subjected to intensive preprocessing routines designed to make them as amenable as possible to deep learning-based modeling. For the tumor type classification task, we retrieved data from a merged and curated repository of two publicly available sources. The first source was the Kaggle Brain Tumor MRI dataset, comprising labeled 2D MRI slices representing four tumor classes: glioma, meningioma, pituitary tumor, and no tumor. The second source was the SciDB Brain Tumor dataset, from which we used additional segmentation annotations that were integrated during preprocessing. The combined dataset derived from the union of several sources greatly added to diversity and overall image quality, in addition to ensuring there was a good balance of tumors throughout the whole set (Figure 1).

Before developing models, thorough preprocessing was carried out on the 2D dataset. All the MRI slices were intensity normalized to the range [0, 1] to reduce inter-scan variations due to acquisition parameters. In addition, Gaussian filtering was used to suppress noise and improve contrast, making tumor areas more visible. The preprocessed images and corresponding segmentation masks were arranged in different directories for training, validation, and testing. The final dataset that was compiled comprised a total of approximately 5000 individual MRI slices, which were evenly distributed across the four various tumor classes under consideration. To split the dataset efficiently and representatively, a stratified data partitioning technique was employed. This technique was used to separate the dataset methodically into training, validation, and test datasets, while still preserving the original class distributions inherent in the data. Specifically, it was determined that 80% of the total data would be available for training, with the remaining 20% being strategically withheld for validation and testing to permit thorough testing. The distributions of every individual partition of the data are clearly depicted in Table 1. It is evident that the data set was quite balanced, which tends to minimize the risk for any probable model bias that could occur in favor of any individual tumor type.

As a preprocessing step for neural network training, all images were resized to 224 × 224 pixels and normalized using ImageNet-standard mean and standard deviation values. Random resizing and random cropping were used as data augmentation techniques during training to enhance model generalization. The 3D tumor segmentation module used the BraTS (brain tumor segmentation challenge) Task01 dataset. The dataset consists of multi-modal MRI scans and pixel-wise tumor segmentation masks for various tumor subregions, such as the tumor core (TC), whole tumor (WT), and enhancing tumor (ET). The dataset was downloaded into the Neuroimaging Informatics Technology Initiative (NIfTI) format, with each sample consisting of image volumes and label masks.

There was a strict preprocessing pipeline applied to the 3D data, including several spatial and intensity normalisation methods to minimise inter-subject variability. Image and label volumes were first affinely registered to the standard RAS coordinate space. Volumes were resampled to 1 mm isotropic resolution with linear interpolation for images and nearest-neighbor interpolation for labels. The images were subsequently intensity normalized on a channel-by-channel basis with non-zero voxel normalization to correct for scanner device and. protocol variations. Spatial data augmentation of the training dataset was performed, comprising random cropping, spatial flipping across all three axes, and small intensity perturbations through random scaling and shifting. Spatial random cropping randomly sampled 128 × 128 × 128 voxel patches of fixed size to fit the GPU memory during training. The augmentation pipeline exposed the models to a huge variety of tumor positions, sizes, and orientations at optimization time. Data was divided into training, validation, and test sets in a ratio of 70-15-15, yielding 338 training instances, 73 validation instances, and 73 test instances. Dataset sizes are given in Table 2.

Apart from total sample numbers, subregion coverage of the tumor was also examined to determine class representation. The whole tumor (WT), tumor core (TC), and enhancing tumor (ET) subregions were well represented within the test and validation sets, where each subset contained complete coverage of all three classes. That said, class imbalance in the training set was moderate in that tumor core, whole tumor, and enhancing tumor regions were found in 311, 326, and 309 training samples, respectively.

**Figure 2:**
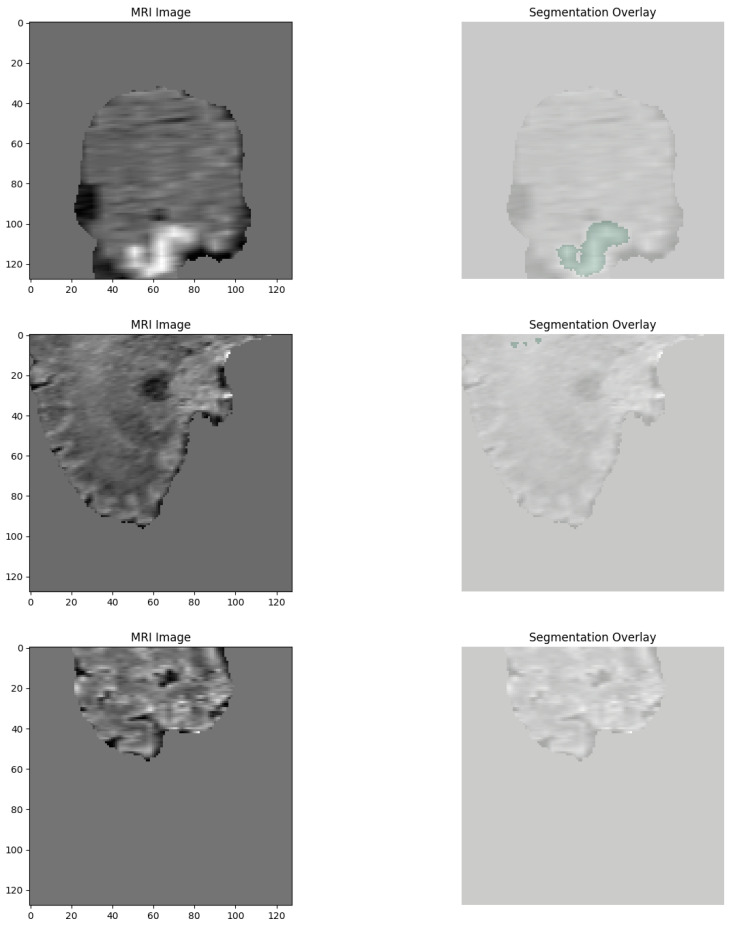
Representative 3D MRI samples with segmentation overlays for TC, WT, and ET.

For better visualization of the dataset structure, a few representative training, validation, and test samples were plotted by randomly extracting slices from the 3D volumes. Segmentation masks of the TC, WT, and ET subregions over the images were coded using red, green, and blue color maps, respectively. A few example images illustrating the anatomical variability and tumor heterogeneity of the dataset are provided in Figure 2.

The thorough preprocessing pipelines detailed in the sections above duly guaranteed high-quality and standardized inputs for use in classification and segmentation model development. This careful strategy also guaranteed the preservation of clinical heterogeneity, which is an inherent component of brain tumor imaging.

### 2.2. Experimental Setup

**Figure 3:**
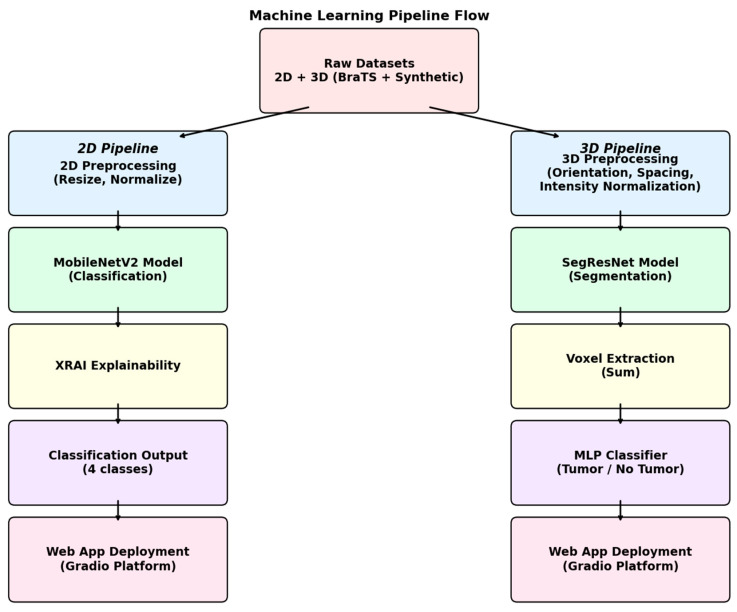
Flowchart of the complete diagnostic pipeline. The system integrates 2D MRI classification, 3D volumetric tumor segmentation, and explainability modules (XRAI saliency maps for 2D, Gaussian-based overlays for 3D). This pipeline is designed for clinical decision support, enabling radiologists to simultaneously obtain predictions, segmentations, and interpretable visual explanations.

The overall experimental pipeline had two distinct but parallel branches, one for the classification of 2D MRI images and the other for segmentation and detection of 3D MRI images. The whole system is a unified combination of various components, like data preprocessing steps, model training, and explainability modules, along with the integration of the entire process in an interactive web application for clinical purposes. The full pipeline architecture is summarized in Figure 3.

**Figure 4:**
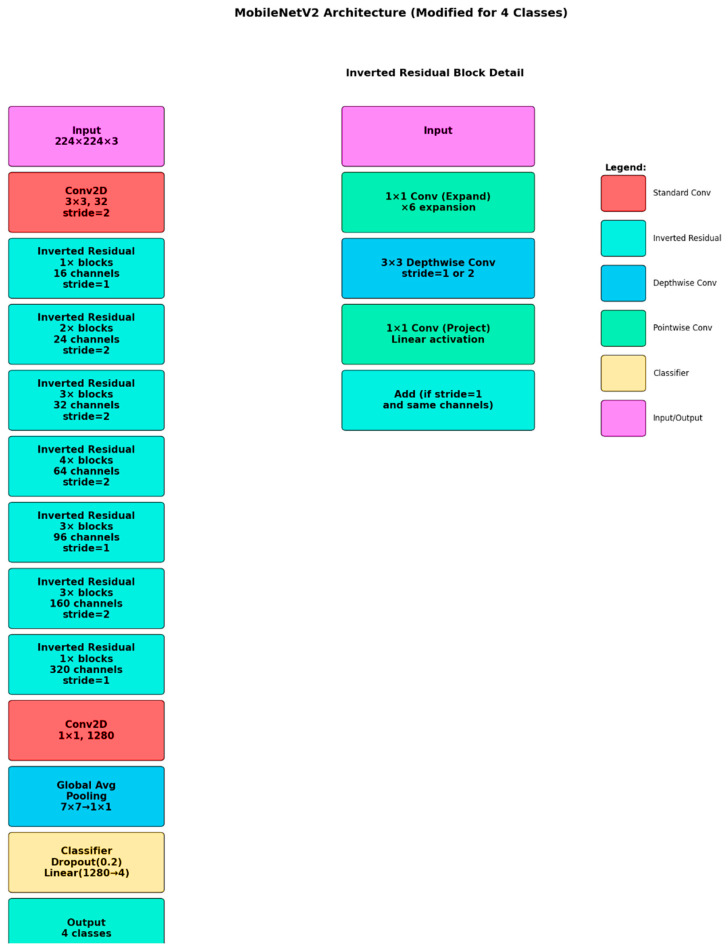
MobileNetV2 architecture.

In order to tackle the 2D brain tumor classification problem, a deep convolutional neural network was designed, with the MobileNetV2 architecture taken as the underlying framework. The model was specifically chosen owing to its lightness in weight, which is one of the factors leading to seamless performance, along with its efficiency and superior suitability in handling smaller medical imaging datasets. The MobileNetV2 base model was pre-trained on the ImageNet dataset with weights, providing us with a solid starting point for our task. In order to fine-tune the model for application in the classification of brain tumors, we carried out the required adjustment of changing the final fully connected layer to a new linear layer. The new layer contains four output neurons, corresponding to each class: glioma, meningioma, pituitary tumor, and the absence of a tumor. For complete comprehension of the structure, the entire architecture is illustrated in Figure 4.

Before training, all 2D MRI slices were resized to 224 × 224 pixels and normalized with ImageNet normalization parameters. For training, the Adam optimizer with a learning rate of 1 × 10^−4^ was used, and cross-entropy loss was taken as the objective function. Training was carried out for 10 epochs on an NVIDIA GPU with model checkpointing on the best validation accuracy achieved.

**Figure 5:**
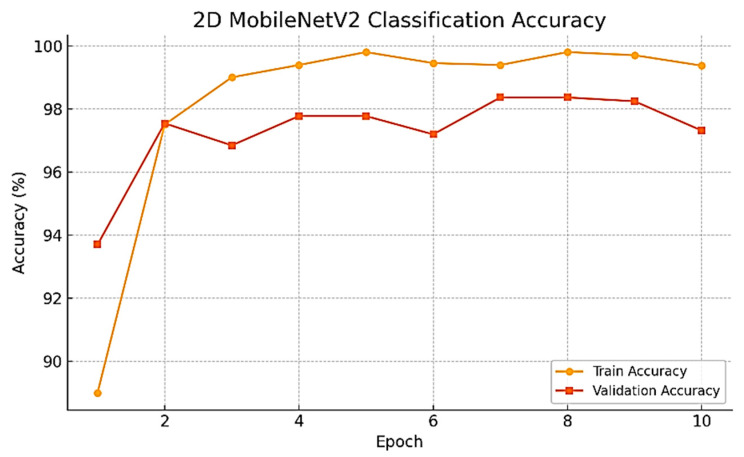
Training and validation accuracy graph.

As the training progressed over time, we observed a series of consistent and noteworthy improvements in the performance of the model being developed. Specifically, the validation accuracy improved dramatically, rising from the initial value of 93.70% during the very first epoch to the remarkable high of 98.37% achieved at epoch 7. Subsequently, the best-performing model was evaluated on the held-out test set, resulting in an impressive test accuracy of 98.02%. To develop a thorough understanding of this improvement, the whole training and validation accuracy curves have been plotted and are presented for review in Figure 5.

**Figure 6:**
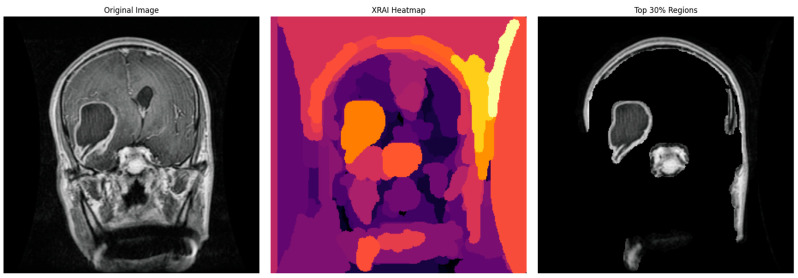
Examples of XRAI saliency maps for 2D classification. The heatmaps highlight regions most influential for the model’s decision. Yellow-to-white regions indicate the highest attribution (most important for the prediction), while orange to red indicate medium attribution levels, and purple regions the lowest attribution levels. These maps enable clinicians to confirm that model decisions correspond to tumor-relevant anatomical areas.

In order to complement and augment the clinical interpretability of the model predictions, the explainability technique named XRAI, or extended region attribution, was incorporated into the system. The new XRAI technique was employed to generate dense saliency heatmaps, which were capable of highlighting the exact spatial regions that contributed most to the model’s classification results. These informative heatmaps, as illustrated in Figure 6, were seamlessly integrated into the deployed clinical user interface, thereby making clear and interpretable justification for all model predictions. For visualizations in the manuscript figures, we displayed the top 30% of attribution pixels to show broader salient regions. In the deployed web application, a stricter threshold (top 5% attribution pixels) was used to reduce noise and emphasize the most relevant tumor regions for clinical use.

**Figure 7:**
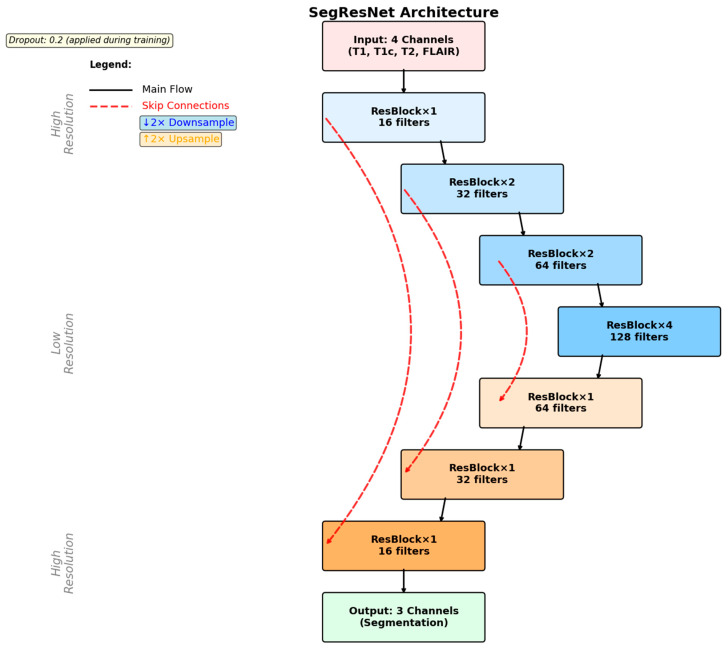
SegResNet architecture.

In order to tackle the difficult task of volumetric tumor segmentation, a 3D convolutional neural network was designed, drawing inspiration from the highly acclaimed SegResNet architecture. Its implementation was based on the high-performance MONAI framework, which is ideally suited for the task at hand in medical imaging. Training of this network was conducted based on multi-modal MRI volumes from the BraTS Task01_BrainTumour dataset, a very popular data resource within the field. Training was conducted specifically for three distinctively different segmentation targets, which play an important role in proper diagnosis and treatment planning: tumor core (TC), whole tumor (WT), and enhancing tumor (ET). Data preparation for this task involved preprocessing of the labels and their formatting to multi-channel binary masks, adhering strictly to the annotation standards provided by BraTS. Furthermore, Figure 7 provides the step-by-step illustration of the SegResNet architecture, thus shedding light on its complicated design and functionality.

All of the 3D volumes were resampled via the procedure of obtaining an isotropic spacing of 1 × 1 × 1 mm. Additionally, reorientation of these volumes was carried out to align with the RAS orientation conventions. Additionally, normalization was also performed using the mean and standard deviation computed specifically over the non-zero voxels within each of the individual channels. Random 3D patches of size 128 × 128 × 128 voxels were randomly cropped for training. In order to further reinforce the generalization and improve the performance of the model, a broad spectrum of data augmentation techniques was implemented. This involved procedures such as flipping images, intensity scale adjustment, and intensity shifting being performed on the dataset.

The training process was conducted with the Dice Loss function, since it’s specifically designed for this task, alongside the Adam optimizer. The optimizer was set to run at a learning rate of 1 × 10^−4^ and the training process extended to over a total of 50 epochs. Throughout the training, the model’s performance kept improving steadily, with incremental gains at each step. By the conclusion of the training, the Dice coefficient achieved an impressive figure of 0.6805 at the final epoch, an achievement that has been shown before and can be observed in Figure 5.

To continue increasing the model’s capacity for generalizing well to new unseen data, synthetic no-tumor volumes were generated through carefully sampling image intensities with a Gaussian distribution and then providing corresponding empty ground-truth masks for these created volumes. A total of 100 of these synthetic volumes was incorporated into the training set, enabling a more varied dataset. After this incorporation, fine-tuning of the model was carried out for another 8 epochs using both the synthetic data and the real data.

The SegResNet model takes as input four MRI channels, T1, T1CE, T2, and FLAIRstacked as separate input channels. These modalities provide complementary anatomical and pathological information: T1 and T1CE highlight structural detail and contrast enhancement, T2 emphasizes edema and cystic components, and FLAIR suppresses cerebrospinal fluid to delineate tumor-associated hyperintensities. By utilizing all four channels simultaneously, the network learns multi-parametric features that enhance tumor localization and boundary delineation. During inference, each voxel in the MRI volume is assigned a probability of belonging to tumor subregions (WT, TC, ET). Non-tumor voxels, including normal brain parenchyma and extra-cranial structures, are suppressed by thresholding these probabilities at 0.5. Consequently, only voxels predicted as tumor remain in the final masks, effectively isolating tumor signal from the rest of the head and brain. These masks are then used for visualization and for extracting volumetric features for the downstream MLP classifier.

In addition to the voxel-wise segmentation process that was followed, a comprehensive meta-classification model was trained with the exclusive intention of predicting the presence or absence of tumors straight from the data that was analyzed. The multilayer perceptron (MLP) classifier employed in this context took carefully extracted voxel count features from the segmentation masks, such as tumor labels of TC, WT, and ET, as model inputs. The MLP model consisted of two hidden layers with 16 neurons in the first hidden layer and 8 neurons in the second hidden layer, respectively. This model underwent an exhaustive training process that consisted of 1000 iterations, where the Adam optimizer was employed to enhance the learning efficiency. The classifier exhibited strong performance measures with excellent precision, recall, and accuracy rates when evaluated on the hold-out test set.

An additional explainability module was developed for the 3D segmentation outputs. After segmentation inference, the predicted WT and ET masks were thresholded at 0.5 probability. Two complementary visualizations were generated in the deployed web application. First, the tumor segmentation overlay displays only the predicted masks on a black background, providing a clear, isolated view of the model’s raw output without anatomical context. Second, the explainability overlay combines the same segmentation masks with the corresponding axial MRI slice, with Gaussian smoothing applied to emphasize high-confidence tumor voxels. This two-panel approach allows clinicians to verify both the exact voxels predicted as tumor (top panel) and their anatomical correspondence on MRI (bottom panel). These overlays confirm that the segmentation output aligns with actual tumor regions in the MRI scan while maintaining interpretability for clinical use.

All the models, classification outputs, segmentations, and explainability modules were incorporated into one interactive web-based application developed using the Gradio framework. The interface accommodates 2D and 3D MRI upload by clinicians, fetching predictions, and rendering of all the explainability visualizations. The whole system facilitates clinical translation and transparent model behavior assessment across tasks.

## 3. Results

**Figure 8:**
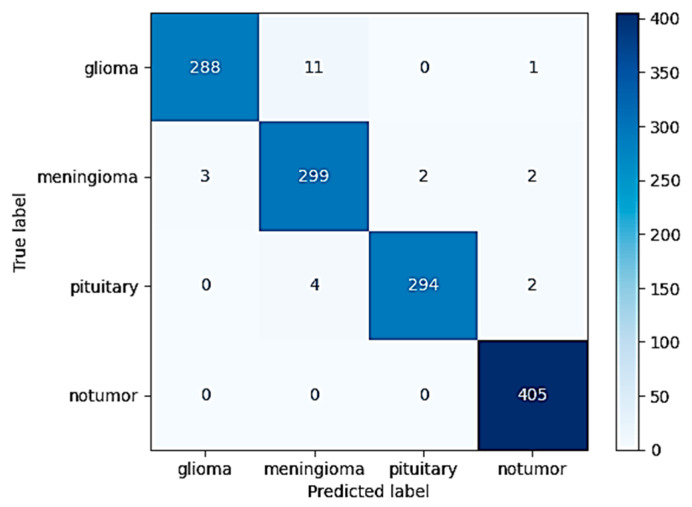
Confusion matrix for 2D test set performance.

The highly optimized MobileNetV2 model demonstrated strong classification performance across the different tumor types considered in the study. When tested on the withheld independent test set, which was used to strictly evaluate its performance, the model was able to attain an excellent overall accuracy of 98.09%. This high accuracy serves to highlight the strong generalization ability of the model in being able to effectively differentiate and classify instances into four diverse classes: glioma, meningioma, pituitary tumor, and instances in which no tumor was even present. The extensive and complete confusion matrix illustrated in Figure 8 evidently reveals that the occurrence of classification errors was minimal. Furthermore, one can notice that these errors took place predominantly among glioma and meningioma classes. This is interpretable both because of the partial anatomical overlap and because of the shared imaging characteristics between such specific subtypes of tumors.

In addition to overall global accuracy metrics, post hoc explainability was also used via the XRAI technique to produce saliency maps for specific predictions. The maps provided a visual inspection of which areas in the MRI scans were most affected by the decision made by the model. An example test set case is included below for each tumor type to enable assessment of the explainability performance.

**Figure 9:**
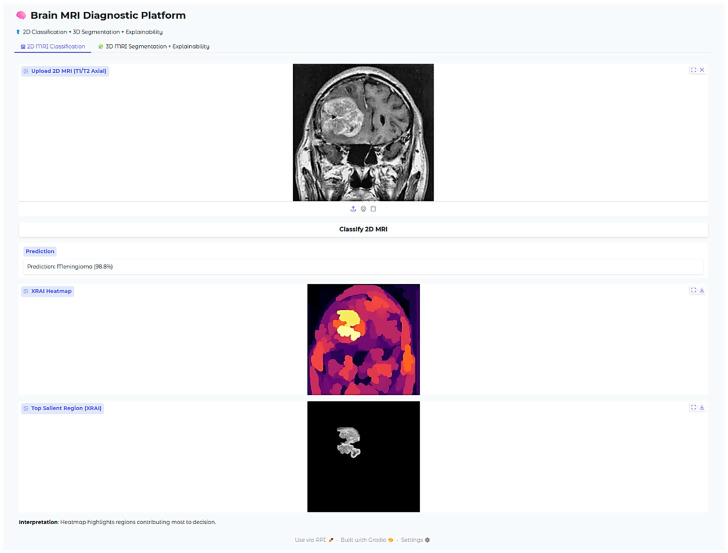
Meningioma classification case (98.8% confidence).

The XRAI saliency map visible in Figure 9 plainly indicates a highly concentrated region ranging from yellow to white. The region is situated in the superior midline parasagittal area and reveals a significant overlap with the actual location of the meningioma. Additionally, the heatmap includes pale yellow and white colors that denote the regions contributing most significantly to the model’s decision made. This exact correlation indicates that the model has been able to demonstrate its ability to concentrate on the common anatomical locations of meningiomas that are situated near the falx cerebri. This is not just an indication of its ability to distinguish between various features to a large degree but also adds validity to its reasoning within the realm of anatomy.

In this case with glioma, then, the saliency overlay is utilized to highlight and call attention to the abnormally hyperintense regions that are present in the deep white matter of the brain, which directly map onto the areas in which the glioma is infiltrating. The light-yellow area present in the center represents a high attribution rate focused on the core of the tumor itself, highlighting its salient characteristics. The orange and red hues present around this area indicate the model’s heightened sensitivity to the presence of peritumoral edema, or fluid accumulation around the tumor. The position of the heatmap shows strong correspondence with the typical infiltrative growth patterns of gliomas, therefore suggesting that the model is, in fact, considering both the morphology of the tumor as well as the extent of edema when it is making its predictions about the behavior and characteristics of the glioma.

**Figure 10:**
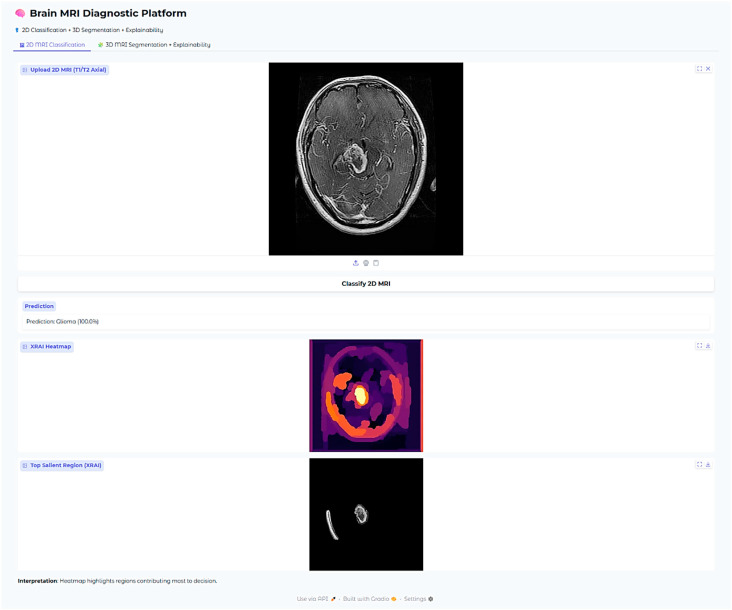
Glioma classification case (100% confidence).

**Figure 11:**
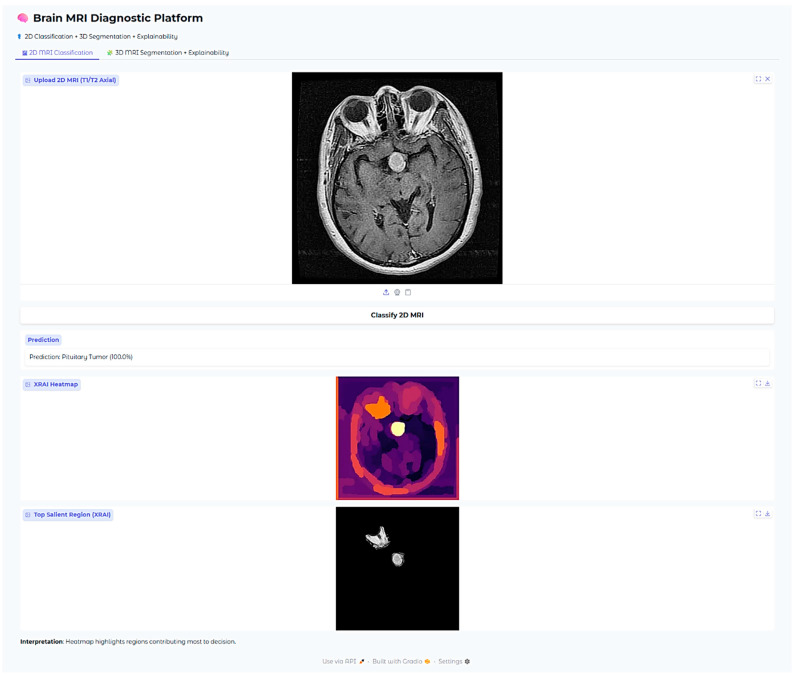
Pituitary tumor classification case (100% confidence).

**Figure 12:**
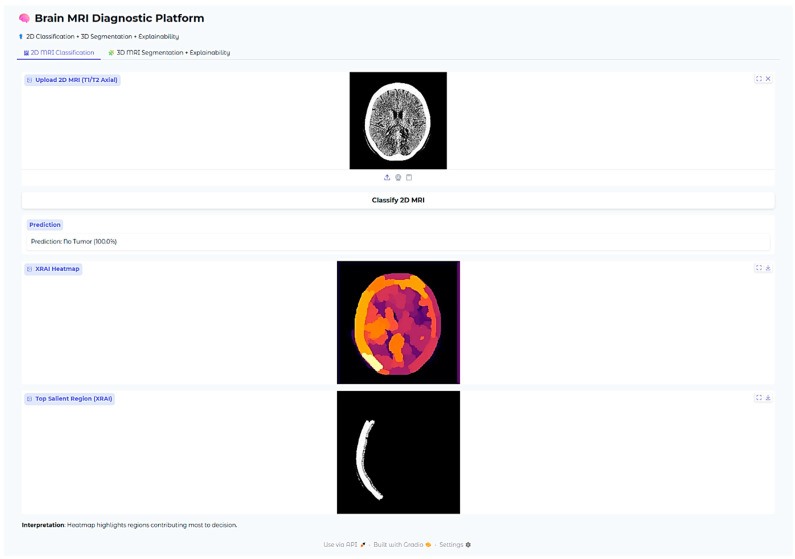
No tumor case (100% confidence).

The Brain MRI Diagnostic Platform showed strong performance on a range of test cases. In one case, the model correctly classified a glioma case with 100% confidence (Figure 10), indicating its strength in identifying malignant brain tumors. Likewise, it correctly identified a pituitary tumor (Figure 11) with 100% confidence, showing its strength in identifying varying tumor types. Additionally, the platform rightly predicted no tumor presence in another test case (Figure 12), indicating its trustworthiness in excluding tumor existence when there is none. These findings cumulatively confirm the model’s proficiency in accurate brain MRI classification.

For pituitary tumors, the XRAI heatmap showed a localized attribution that was precisely positioned directly over the sellar area, a critical area for such tumors. The intensely visible bright yellow activation of the heatmap is precisely coincident with the anatomical site of the pituitary adenoma, which is located at the midline base of the skull. This is also consistent with the radiological appearances typically seen in both sellar and suprasellar tumor masses.

Furthermore, this clear anatomical focus shown by the heatmap is evidently utilized to illustrate the model’s incredible ability to accurately localize tumor regions that are limited, which is crucially important in the effective detection and diagnosis of pituitary tumors. The model’s focus on clinically significant anatomical detail determines its potential value as an adjunct to neuroradiological practice, most especially for aiding diagnostic precision and minimizing interpretive variation.

In the no-tumor scenario, the XRAI saliency map shows a low-intensity, diffuse heatmap over the brain parenchyma. The absence of any concentrated bright yellow or white region suggests that the model had not detected any abnormal regions.

This is reassuring that the model correctly suppresses non-pathologic features and does not show false positive activations in the context of healthy brain scans.

Operationally, the entire 2D classification pipeline, explainability generation therein, was consuming 30 to 40 s per scan, which made the system feasible for interactive use in a real-time web interface.

The 3D segmentation experiment was carried out with the SegResNet model trained on the augmented BraTS dataset with 100 synthetic no-tumor cases for enhanced robustness. The top-performing model obtained a mean Dice similarity coefficient of around 68% for tumor subregions. After fine-tuning with the 100 synthetic no-tumor volumes for an additional 8 epochs, the Dice coefficient further improved, reaching a final value of 0.7057.

The inclusion of synthetic no-tumor volumes reduced the model’s tendency to produce false positives in cases without visible tumors by exposing the network to a broader variety of normal brain appearances. This helped the model better distinguish pathological tissue from normal structures. The effect of this augmentation was reflected in the downstream MLP classifier, which achieved 100% specificity for tumor absence on the hold-out test set. This confirms that adding synthetic no-tumor data improved the pipeline’s ability to suppress false positives while maintaining segmentation performance.

Although some segmentation boundary irregularity was present, the final masks were sufficiently accurate for high-level tumor detection. To introduce additional robustness, tumor subregion volume extraction (total WT, ET, TC voxels) was employed to train the downstream MLP binary tumor presence classifier. The classifier yielded 100% test set accuracy (precision, recall, and F1-score all 1.0), reflecting outstanding performance for this clinically relevant task.

**Figure 13:**
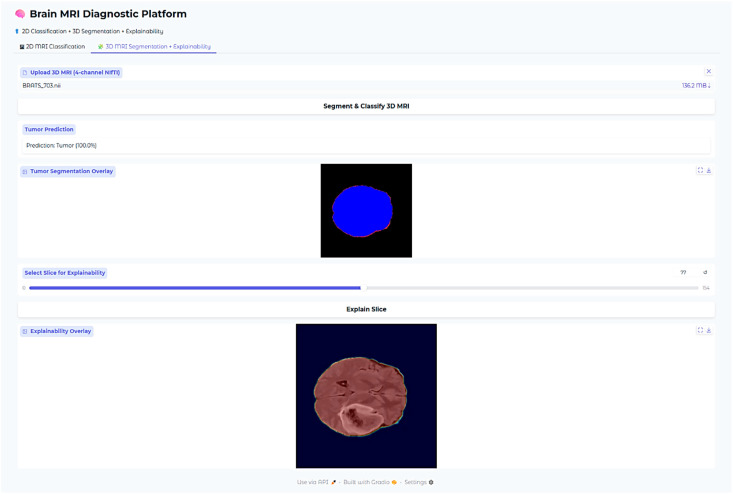
Three-dimensional Case 1.

**Figure 14:**
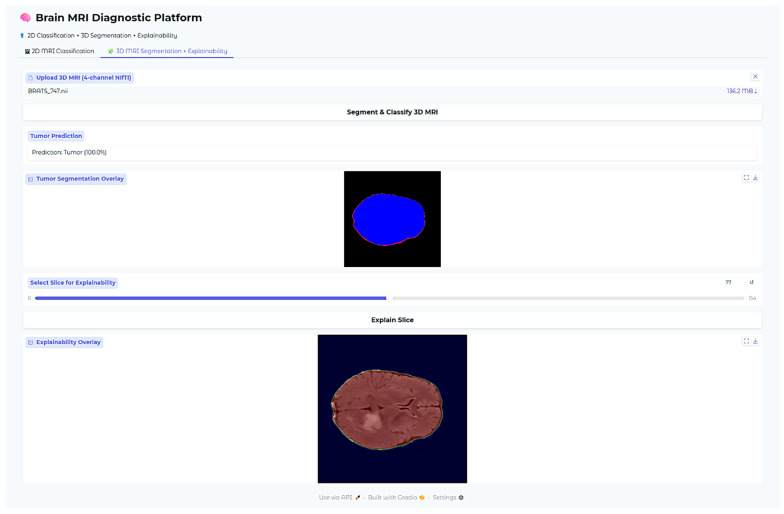
Three-dimensional Case 2.

**Figure 15:**
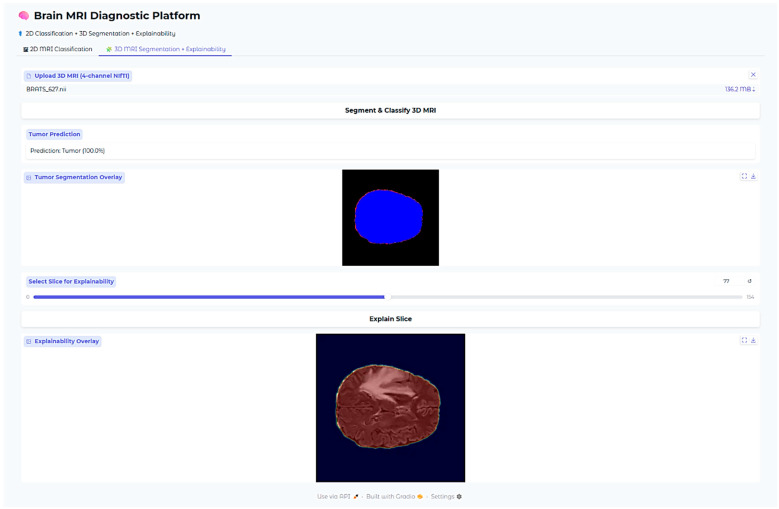
Three-dimensional Case 3.

Explainability of the 3D segmentation model was also assessed using slice-level over-lays that were obtained by Gaussian-smoothing the WT and ET segmentation masks. Representative images are presented in Figure 13, Figure 14 and Figure 15, demonstrating both segmentation quality and spatial explainability.

In Figure 13, the segmentation output successfully localizes the tumor region. The corresponding explainability overlay displays brighter reddish intensities concentrated near the inferior and lateral tumor margins. These brighter areas represent regions where the segmentation model exhibited the highest voxel-wise activation strength (post-sigmoid output near threshold). The alignment between the segmentation mask and the explainability overlay indicates that the model consistently identified these tumor regions as contributing strongly to its segmentation decision.

In Figure 14, we can once more observe the same pattern, which strongly resembles previous findings, with the segmentation map cleanly delineating the bulk of the tumor, defining its presence in explicit terms. Additionally, the explainability overlay performs a crucial role by bringing to light the high-response areas that are observed along the border core of the tumor itself. The areas marked in the brightest shade of red once again indicate voxel clusters that are associated with the greatest degree of model confidence in terms of describing the tumor. The adjacent brain parenchyma is largely dark in the attribution map, indicating a low saliency signal and thereby confirming the model’s capability to spatially limit its predictive confidence to the tumor boundary. Low attribution outside the edge of the lesion is an indicator that the model is specific and can suppress false positives in the non-pathological tissue neighboring the lesion.

In Figure 15, the overlay highlighting explainability demonstrates a drastically sharper and more accurate localization, with clusters that are vivid yellowish-red and closely placed near the center of the tumor. This serves to reinforce that the model is consistently focusing its activation on the pertinent subregions of the tumor in different patients, pointing to a high degree of reliability in its operation. Most notable is that, even though there is an outer segmentation mask that looks overall rough and a bit noisy as a result of the nature of voxel-wise thresholding, the heatmaps relating to explainability offer well-refined visual indications. These indications specifically demonstrate the zones that the model has most confidently segmented, thus providing us with a better grasp of its analytical properties.

Despite the fact that the raw segmentation boundaries at times seem irregular and rough because of the inherent characteristics that come with thresholding the outputs of probabilistic models, the overlays that are used to provide explainability serve to add and elaborate on the interpretation of the results. This is due to the fact that by accentuating specific areas where the model indicates high confidence in its identification of tumors, the overlays provide further insights besides imparting visual confidence that adds further conviction to clinicians when reviewing and assessing the results that are generated by automated segmentation techniques.

## 4. Discussion

The current study proposes an end-to-end, complete acquired brain tumor classification system with two-dimensional (2D) multi-class classification and three-dimensional (3D) segmentation, as well as strong explainable artificial intelligence (XAI) methods. The entire pipeline was designed with a keen eye on both clinical utility and real-world deployment readiness, with particular attention to usability by radiologists, neurosurgeons, and healthcare professionals in actual everyday neuro-oncology clinical workflows.

The 2D classification module was very successful at distinguishing between glioma, meningioma, pituitary tumor, and no tumor. The MobileNetV2 backbone pre-trained on the clean dataset reached 98.09% test accuracy on the test cohort. This strong performance was largely attributed to various synergistic design choices, including the employment of transfer learning from ImageNet, the use of effective preprocessing and data augmentation, and careful class balance management throughout dataset construction. The model’s ability to generalize was proven in all tumor classes without substantial performance loss in any subgroup, in favor of its potential to be applied in a wide range of clinical cases.

Aside from accuracy, XRAI explainability was crucial for clinical proof of model behavior. XRAI was selected as the explainability technique because it produces region-based saliency maps that highlight contiguous areas of interest, which are more clinically interpretable for radiologists compared to pixel-level or fragmented heatmaps. Methods such as Grad-CAM can produce coarse and often discontinuous regions, while LIME and SHAP are perturbation-based and do not inherently preserve spatial structure, making them less suitable for medical imaging tasks. XRAI, being based on integrated gradients with region attribution, is particularly effective for medical images where anatomical consistency is important. Although we did not conduct a direct comparison with other XAI techniques in this study, since our focus was on developing a complete 2D–3D diagnostic pipeline, future work will involve a systematic evaluation of XRAI against Grad-CAM, LIME, SHAP, and related techniques using the same dataset to ensure fair comparison. Saliency maps produced by XRAI consistently emphasized tumor-relevant anatomical areas on the MRI scans. That is, yellowish and whitish highlighted regions in saliency maps generally overlapped with the actual sites of tumors, as visually verified in instances of glioma, meningioma, pituitary tumor, and no tumor predictions. This alignment between the model’s attention and true pathological areas ensures that the model is making its decisions based on valid imaging biomarkers instead of superficial image features or dataset artifacts. Such interpretable decision-making is important to both clinician trust as well as downstream regulatory pathways for AI adoption in the clinic. In addition, these XAI explanations and classification predictions were attained in 30–40 s per patient case, rendering the system feasible for near real-time clinical applications.

For 3D tumor segmentation, we used a SegResNet architecture to take in multi-modal volumetric MRI inputs. After initial training and fine-tuning stages, the model converged at Dice similarity coefficients between 68% and 70% for both the validation and test datasets. Although numerically inferior to leading models on highly annotated challenges such as BraTS, this is still very suitable in light of a number of factors. First, the training set size captures realistic data availability in hospital environments, where hundreds of labeled MRI volumes rather than thousands are the norm. Second, the addition of 100 synthetic no-tumor volumes to the training data added class diversity required for trustworthy tumor screening deployments but could add variability per se at the voxel boundary level. Third, the system was trained end-to-end on multi-modal 4-channel input volumes directly without extreme modality-specific pretraining or lengthy handcrafted post-processing pipelines, with generalizability and robustness being the focus. Lastly, resource-efficient patch-wise cropping was utilized, enabling training on modest hardware while still being able to capture volumetric tumor morphology.

The BraTS dataset used in this study contains four standard MRI sequences (T1, T1CE, T2, and FLAIR) but does not include diffusion-weighted or perfusion imaging. Future studies using multi-parametric MRI datasets that incorporate these additional modalities could provide richer physiological information and potentially improve tumor boundary delineation and model robustness.

Notably, though Dice scores reflect voxel-level mask alignment, they do not convey the full clinical value of the segmentation outcomes. Visual inspection of the 3D explainability overlay showed that even where segmentation borders were slightly irregular, the model accurately localized dense voxel regions of the tumor. Gaussian-weighted overlays systematically accentuated dense tumor centroids that correlated well with the most clinically pertinent regions for surgical planning, radiotherapy planning, and disease burden estimation. For the three example test cases shown, heatmaps were highly sensitive to the presence of actual tumors, which vindicates the system’s usefulness for real-world diagnostics. The slightly rough boundary artifacts are likely caused by thresholding hard probability maps to create binary masks, rather than genuine model uncertainty during tumor localization.

In order to enhance the clinical usefulness and efficiency of the platform even more, an entire two-stage hybrid classification procedure was successfully conducted. This was carried out by training an MLP meta-classifier on volumetric features that are specifically extracted from the segmentation masks that are utilized in analysis. The volumetric features were successfully obtained by summing up the voxels of the various tumor subregions, which include TC, WT, and ET. With this process, precise and consistent binary detection of tumors has been possible, leading to improved diagnostic outcomes. The MLP classifier worked impeccably well on the test set with an astounding accuracy of 100%, and achieved 100% precision, recall, and F1-score. This second phase of the classification process can enable rapid and efficient screening for the presence or absence of tumors at a high level, which can be utilized as an important first triaging tool in routine radiological practices found in day-to-day clinical activities.

All of the system elements were finally deployed to one interactive web application platform via Gradio. The interface supports both 2D and 3D data processing with real-time monitoring of predictions, segmentations, and attendant explainability heatmaps. The system design is suitable for research prototyping as well as for future possible clinical deployment scenarios, providing fast, interpretable, and user-interpretation AI assistance directly available to radiologists and clinicians. Whereas 2D predictions took less than 40 s, full 3D segmentation usually required about 30 s, including loading the uploaded NIfTI file, converting it to the model input tensor, running inference, and generating the visualization. The reported inference time excludes preprocessing operations such as resampling and normalization, which were applied during training but omitted in the web demo because the BraTS dataset is already co-registered and roughly standardized. In a real clinical setting, additional preprocessing would be required for raw MRI volumes, which would add some extra time but not significantly impact the overall usability of the pipeline. While inference on the BraTS dataset was feasible without extra preprocessing in the deployed web application, real-world clinical MRI data would require additional steps such as skull stripping, bias field correction, resampling, and scanner-specific intensity normalization. Future work will integrate these preprocessing steps and validate the pipeline on external, multi-center datasets to ensure robustness across scanners and acquisition protocols.

In spite of the demonstrated performance and clinical promise, several limitations must be considered. Both segmentation and classification models were developed and evaluated on publicly available benchmark datasets, which may not capture the heterogeneity of actual clinical populations in the setting of multi-center and multi-scanner variability. External validation studies must be performed prior to clinical deployment of these models. Future work will involve external validation on multi-center datasets, incorporating MRI scans from different scanners and acquisition protocols. This would include domain adaptation to account for scanner-specific intensity variations and prospective testing within hospital workflows in collaboration with clinicians. Such validation will be essential to confirm the system’s robustness and usability in real clinical environments.

In addition, while the segmentation model was good at delineating tumor regions, there were certain irregularities at boundaries. Future improvements could involve post-processing techniques such as conditional random fields for enforcing spatial mask accuracy, uncertainty quantification, or employing boundary-aware loss functions.

Finally, while the saliency maps provided valuable visual insights, our evaluation of their alignment with tumor regions was qualitative rather than quantitative. This was because segmentation masks were not available for all images in the 2D classification dataset. In future studies, when paired tumor masks are available, we plan to compute quantitative metrics such as intersection-over-union (IoU) or pointing game scores to objectively measure how well saliency maps correspond to pathological regions. In addition, we observed that XRAI occasionally highlights non-brain regions, particularly near image boundaries where intensity gradients are high (see Figure 6). While these regions are generally suppressed when only the top 30% of attribution pixels are visualized, even stricter thresholds (e.g., top 20% or 10%) could further reduce irrelevant saliency, which the web application does by showing the top 5%. Future work may also involve applying skull-stripping or brain masking before attribution computation to remove non-brain signals while retaining tumor visibility. Such refinements could enhance the clinical interpretability of saliency maps without diminishing their diagnostic value.

## 5. Conclusions

In this detailed study, a complete and thorough end-to-end diagnosis system tailored to brain tumors was carefully developed. This new system integrates both two-dimensional (2D) multi-class classification techniques as well as three-dimensional (3D) segmentation models, all within a common and integrated explainable artificial intelligence (XAI) framework. The 2D MobileNetV2-based classifier, the backbone of this system, achieved an impressive test accuracy rate of 98.09% in distinguishing between different types of tumors like glioma, meningioma, pituitary tumors, and the absence of any tumor types. Conversely, the 3D SegResNet segmentation model, another vital part of this research, achieved Dice similarity coefficients ranging approximately between 68% to 70% after undergoing initial training as well as synthetic fine-tuning processes. A multilayer perceptron classifier, which is an assistive technology that effectively utilizes volumetric segmentation features, has also facilitated the task of binary tumor detection at a high accuracy rate of 100%.

The application of post hoc explainability methods, namely XRAI saliency maps natively designed for 2D classification tasks, in conjunction with Gaussian-weighted overlays amenable to 3D segmentation tasks, has resulted in the generation of concise, transparent, and interpretable visual results that shed light on the decision-making processes applied by the model. Moreover, all of the elements comprising this novel approach were effectively applied in an interactive web-based interface, thereby proving both its clinical feasibility and its potential for real-time availability, which holds broad implications for use in a variety of applications across the spectrum of radiology.

In conclusion, the findings of the current study evidently show that the integration of segmentation, classification, and explainability in a single, sole modular framework can provide significant and meaningful clinical support to the diagnosis of brain tumors. Furthermore, the novel approach represents a solid foundation for future large-scale prospective validation and deployment possibilities in various centers.

## Figures and Tables

**Table 1:** Class distribution for 2D classification dataset.

Subset	Glioma	Meningioma	Pituitary	No Tumor	Total
**Training**	1123	1137	1247	1348	4855
**Validation**	198	202	210	247	857
**Test**	300	306	300	405	1311

**Table 2:** Three-dimensional dataset partition sizes.

Subset	Number of Samples
**Training Set**	338
**Validation Set**	73
**Test Set**	73

## Data Availability

Data are contained within the article.
